# Biochemical Analysis of Two Single Mutants that Give Rise to a Polymorphic G6PD A-Double Mutant

**DOI:** 10.3390/ijms18112244

**Published:** 2017-10-26

**Authors:** Edson Jiovany Ramírez-Nava, Daniel Ortega-Cuellar, Hugo Serrano-Posada, Abigail González-Valdez, America Vanoye-Carlo, Beatriz Hernández-Ochoa, Edgar Sierra-Palacios, Jessica Hernández-Pineda, Eduardo Rodríguez-Bustamante, Roberto Arreguin-Espinosa, Jesús Oria-Hernández, Horacio Reyes-Vivas, Jaime Marcial-Quino, Saúl Gómez-Manzo

**Affiliations:** 1 Laboratorio de Bioquímica Genética, Instituto Nacional de Pediatría, Secretaría de Salud, Mexico City 04530, Mexico; edsonjiovany@ciencias.unam.mx (E.J.R.-N.); jesus.oria.inp@gmail.com (J.O.-H.); hreyesvivas@yahoo.com.mx (H.R.-V.); 2 Laboratorio de Nutrición Experimental, Instituto Nacional de Pediatría, Secretaría de Salud, Mexico City 04530, Mexico; dortegadan@gmail.com; 3 Consejo Nacional de Ciencia y Tecnología (CONACYT), Laboratorio de Agrobiotecnología, Tecnoparque CLQ, Universidad de Colima, Carretera los Limones-Loma de Juárez, Colima 28629, Mexico; hjserranopo@conacyt.mx; 4 Departamento de Biología Molecular y Biotecnología, Instituto de Investigaciones Biomédicas, Universidad Nacional Autónoma de México, Mexico City 04510, Mexico; abigaila@correo.biomedicas.unam.mx; 5 Laboratorio de Neurociencias, Instituto Nacional de Pediatría, Secretaría de Salud, Mexico City 04530, Mexico; america_vc@yahoo.com.mx; 6 Laboratorio de Inmunoquímica, Hospital Infantil de México Federico Gómez, Secretaría de Salud, Mexico City 06720, Mexico; beatrizhb_16@comunidad.unam.mx; 7 Colegio de Ciencias y Humanidades, Plantel Casa Libertad, Universidad Autónoma de la Ciudad de México, Mexico City 09620, Mexico; edgar.sierra@uacm.edu.mx; 8 Departamento de Infectología e Inmunología, Instituto Nacional de Perinatología, Secretaría de Salud, Mexico City 11000, México; jesspinq@yahoo.com.mx; 9 Departamento de Química de Biomacromoléculas, Instituto de Química, Universidad Nacional Autónoma de Mexico, Mexico City 04510, Mexico; e-rodriguez-bustamante@ciencias.unam.mx (E.R.-B.); arrespin@unam.mx (R.A.-E.); 10 Consejo Nacional de Ciencia y Tecnología (CONACYT), Instituto Nacional de Pediatría, Secretaría de Salud, Mexico City 04530, Mexico

**Keywords:** G6PD deficiency, kinetic parameters, thermal stability

## Abstract

Glucose-6-phosphate dehydrogenase (G6PD) is a key regulatory enzyme that plays a crucial role in the regulation of cellular energy and redox balance. Mutations in the gene encoding G6PD cause the most common enzymopathy that drives hereditary nonspherocytic hemolytic anemia. To gain insights into the effects of mutations in G6PD enzyme efficiency, we have investigated the biochemical, kinetic, and structural changes of three clinical G6PD variants, the single mutations G6PD A+ (Asn126AspD) and G6PD Nefza (Leu323Pro), and the double mutant G6PD A− (Asn126Asp + Leu323Pro). The mutants showed lower residual activity (≤50% of WT G6PD) and displayed important kinetic changes. Although all Class III mutants were located in different regions of the three-dimensional structure of the enzyme and were not close to the active site, these mutants had a deleterious effect over catalytic activity and structural stability. The results indicated that the G6PD Nefza mutation was mainly responsible for the functional and structural alterations observed in the double mutant G6PD A−. Moreover, our study suggests that the G6PD Nefza and G6PD A− mutations affect enzyme functions in a similar fashion to those reported for Class I mutations.

## 1. Introduction

Glucose-6-phosphate dehydrogenase (G6PD, EC 1.1.1.49) is a metabolic enzyme that catalyzes the first and rate-limiting step of the pentose phosphate pathway (PPP). This enzyme participates in glucose-6-phosphate conversion to 6-phosphoglucono-δ-lactone with the concomitant production of a reduced form of nicotinamide adenine dinucleotide phosphate (NADPH) [1,2]. G6PD has a key role in the regulation of NADPH cell levels, which is a main component in the erythrocytes against oxidative damage.

Given the central role of G6PD in the regulation of PPP, mutations in this gene have important phenotypic consequences. Heterozygous inactivating mutations in the *G6PD* gene can cause acute hemolytic anemia [3], characterized by several anemia, jaundice, and splenomegaly [4,5]. A total of 217 mutations in the G6PD gene have been described, which are mainly located in the coding regions and are buried in the enzyme producing, functionally-deficient G6PD variants [6]. Most of the reported G6PD variants are due to single-point mutations causing single amino acid substitution; however, double and triple mutants have also been reported, but with lower frequencies [6,7].

From the 217 G6PD mutations reported worldwide, Mexico has reported the existence of 19 different mutants, including the single-point mutations G6PD A+ (Asn126Asp) and G6PD Nefza (Leu323Pro), which has given rise to the double mutant G6PD A− (Asn126D + Leu32Pro) that has not been functionally and biochemically characterized. Biochemical study of these single natural variants as well as the double mutant G6PD A− is required to fully understand the molecular mechanisms underlying the observed clinical manifestations.

**Figure 1:**
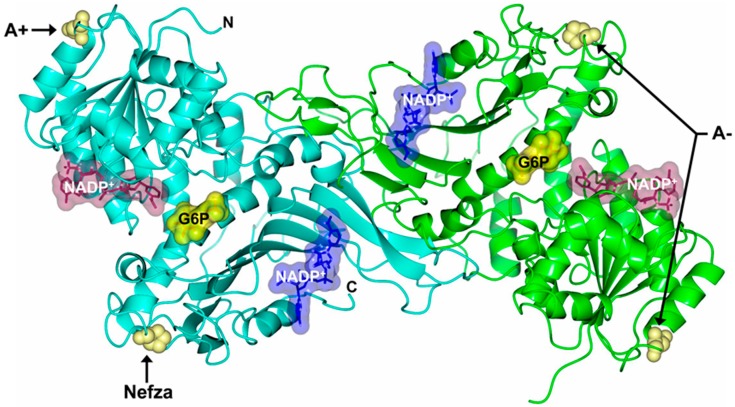
Structure of human Glucose-6-phosphate dehydrogenase (G6PD) dimer (Protein Data Bank entries 2BHL and 2BH9) indicating the location of Class III mutations Nefza (L323P), A+ (N126D), and A− (N126D + L323P) (yellow spheres). Structural nicotinamide adenine dinucleotide phosphate (NADP^+^), catalytic NADP^+^, and glucose-6-phosphate (G6P) are drawn as blue, dark purple, and yellow molecular surface representations, respectively. Monomers are shown in cyan and green. The same color code is used in all other figures.

The single natural Class III G6PD A+ variant involves the substitution of adenine by guanine (A→G) in the nucleotide (nt) 376 (exon 5) with a change at position 126 of asparagine to aspartic acid (Asn→Asp) amino acid residue and is related to a form of asymptomatic G6PD deficiency [8,9]. The single natural Class III G6PD Nefza variant was detected in an 18-year-old man from Tunisia with antecedents of hemolytic anemia triggered by bean consumption. This mutant involves a substitution of thymine for cytosine (T→C at nt 968 (exon 9), which results in the replacement of the amino acid leucine by proline 323 (Leu→Pro), located at a distance of ~9 Å from the substrate-binding site for β-d-glucose-6-phosphate (G6P) (Figure 1). The double mutant G6PD A− (A376G/T968C) involves the mutations G6PD A+ and G6PD Nefza [10,11] (Figure 1). This double mutant is classified as a Class III variant because the patients showed episodes of hemolysis triggered by infections, drugs, or food [12]. Furthermore, it was observed that the patients with this double mutant showed a residual glucose-6-phosphate dehydrogenase activity around 10–20% [13,14].

In this study, we characterized the clinical G6PD variants G6PD A+ and G6PD Nefza to investigate the cooperation of these two single mutations in the double G6PD A− variant. Three natural mutants were constructed, overexpressed, and purified. Detailed steady state kinetics and thermostability assays were carried out and compared with the corresponding values of the human wild type (WT) G6PD enzyme. Finally, from the solved three-dimensional structure of the human G6PD protein, we defined changes in the protein structure to explain the clinical manifestations of these mutations.

## 2. Results and Discussion

### 2.1. Construction, Expression, and Purification of Recombinant G6PD Variants

**Figure 2:**
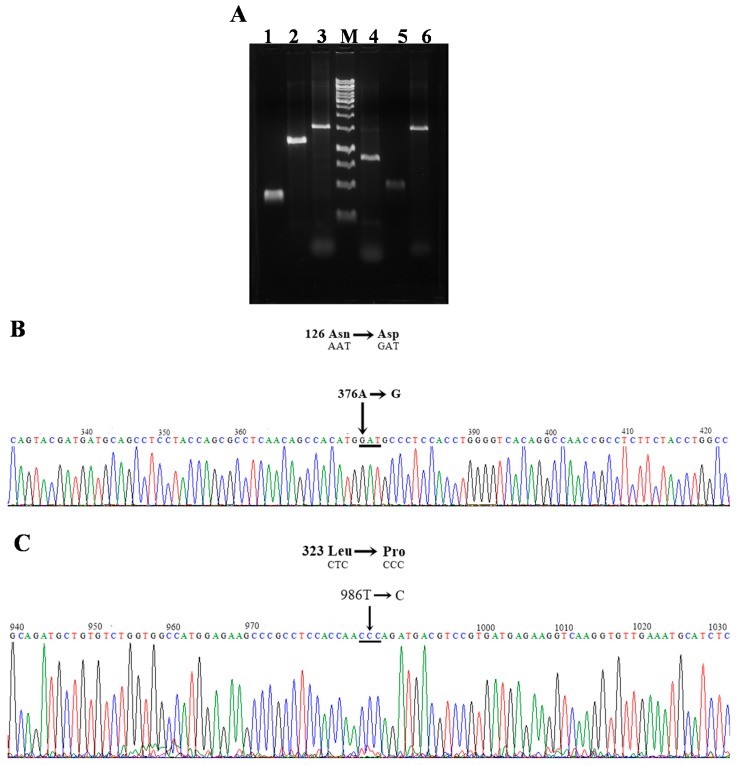
Construction of three clinical G6PD mutants. (**A**) The polymerase chain reaction (PCR) amplified product from the first and second round in agarose gel electrophoresis (1%). M: Marker O´GeneRuler 1 kb DNA ladder (Thermo Scientific). Lane 1, G6PD A+ fragment 1; lane 2, G6PD A+ fragment 2; lane 3, amplification of the full-length G6PD G6PD A+; lane 4, G6PD Nefza fragment 1; lane 5, G6PD Nefza fragment 2; lane 6, amplification of the full-length G6PD Nefza; (**B**) Electropherogram of the single mutant G6PD A+ (A376G, Asn→Asp); (**C**) Electropherogram of the single mutant G6PD Nefza (T968C, Leu→Pro). In all cases the mutations with corresponding nucleotide and amino acid substitutions are shown.

The plasmid pET-HisTEVP-*G6PD* vector expression [15] that contains the full human *G6PD* gene (NM_001042351 access) was used as a template to create two single mutants, G6PD A+, G6PD Nefza, and one double mutant G6PD A− of clinical importance (Figure 2A). Bidirectional DNA sequence analysis of constructed plasmids (pJETg6pd A+, pJETg6pd Nefza, and pJETG6PD A−) was performed to confirm that the desired point mutations were achieved.

**Figure 3:**
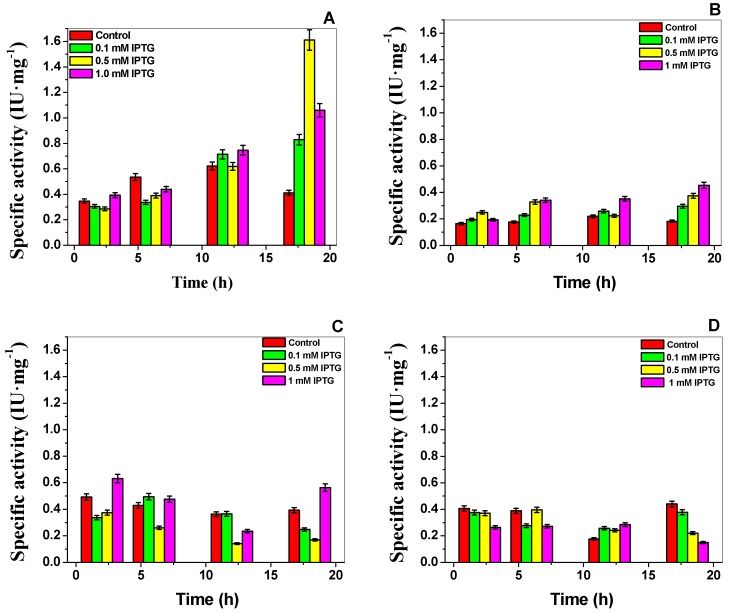
Expression assays of G6PDs in *Escherichia coli* BL21(DE3)Δ*zwf* ::*kanr^r^*. (**A**) Expression of human WT G6PD, two single mutants Class II; (**B**) G6PD A+; (**C**) G6PD Nefza; and (**D**) one double mutant Class II G6PD A−. Specific activity was measured in the crude extract and was used as indicative of the expression levels of soluble recombinant protein. Statistical data were obtained from the three assays.

Recombinant G6PD mutants with the desired mutations (Figure 2B,C) were sub-cloned into the pET-3a plasmid and transformed into competent *E. coli* BL21(DE3)Δ*zwf*::*kanr^r^* to produce large quantities of soluble recombinant protein. We determined the optimal expression conditions of soluble proteins using specific activity as an indicator. We found that while WT G6PD expression was induced by isopropyl-β-d-thiogalactopyranoside (IPTG) in a time and concentration dependent manner, all mutant specific activities were between 50–80% less when compared to the control WT G6PD in the crude extract (Figure 3). For the G6PD A+ and G6PD Nefza mutants, we observed the best specific activity (0.5 IU·mg^−1^ and 0.55 IU·mg^−1^, respectively) using 1 mM IPTG and 18 h of incubation (Figure 3B), which represented a decrease of 3-fold in the specific activity with respect to the WT-G6PD enzyme (Figure 3C). For the double mutant G6PD A−, we obtained an activity of 0.4 IU·mg^−1^ (0.1 mM IPTG and 18 h of incubation), which represented a decrease of 4-fold with respect to the WT-G6PD enzyme (Figure 3D). Even though the studied mutations are reported as Class III variants and are located in different parts of the three-dimensional structure of the WT-G6PD protein, the quantity of purified protein obtained was 50–89% lower than that obtained for WT-G6PD (1.6 IU·mg^−1^) [15] (Figure 3A).

To gain an insight on the effect of the three mutations on the 3-dimensional structure of WT G6PD, we purified the recombinant human G6PDs (WT and mutants) by affinity and anion exchange columns. Sodium dodecyl sulfate-polyacrylamide gel electrophoresis (SDS-PAGE) analysis of purified proteins showed a single band and 96% purity, which allowed us to conduct functional and structural assays. The purification results are summarized in Table 1. The chromatographic steps resulted in a 3.5–4.8 mg of pure protein (per 2 liter of *E. coli* culture); however, purification efficiency for mutants was lower with respect to the WT G6PD enzyme. For mutants G6PD A+, Nefza, and A− variants, the yield values of the purified proteins were of 43, 28, and 24%, respect to total enzyme in the crude extract.

As observed, the purification yield of the Class III G6PD A+ was very similar to that obtained for the WT-G6PD enzyme; while for Class III G6PD Nefza and G6PD A−, yield was much lower. Despite these mutations being reported as Class III variants and the use of optimal conditions for expression, the specific activities in the crude extract and the purification yields among them were different. The results suggested that even though the mutations were located in different regions of the three-dimensional structure and were distant from the active site or from the structural NADP^+^ region, all exhibited a negative effect on G6PD expression. This effect could be related to the lower stability of the mutant proteins affecting catalytic activity of the enzyme.

### 2.2. Measurement of Steady-State Kinetic Parameters

**Figure 4:**
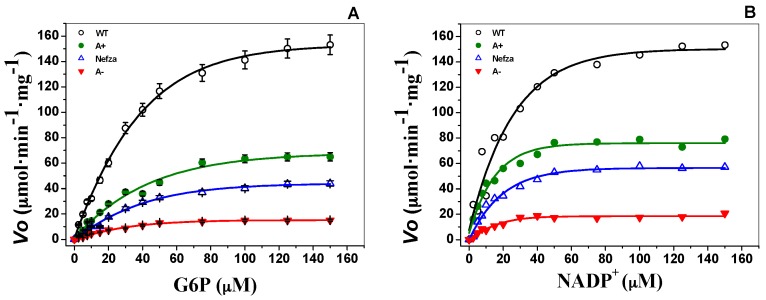
Representative kinetic plots of WT G6PD and three clinical G6PD mutants for (**A**) G6P and (**B**) NADP^+^. Initial velocity (*V*o) data obtained from initial-rate measurements varying one substrate concentration indicated in the abscissa axis with the second substrate fixed at saturating concentration were fitted to the Michaelis–Menten equation by non-linear regression calculations. The data represent mean ± SD from three independent experiments.

To evaluate the effect of mutations on catalytic efficiency, steady-state kinetic parameters were analyzed for the three G6PD variants and wild type by using different concentrations of G6P and NADP^+^. Initial velocity values obtained at the substrate concentrations (indicated in the abscissa axis) were fitted to the Michaelis–Menten equation by non-linear regression calculations (Figure 4). Steady-state kinetic parameters were obtained from plots and are summarized in Table 2. The K_m_ values for the Class III G6PD A+ (K_m_G6P = 56.4 ± 5.5, K_m_NADP^+^ = 12.9 ± 1.4 μM) and G6PD Nefza (K_m_G6P = 50.4 ± 6.2, K_m_NADP^+^ = 16.4 μM) mutants were about twice as high as that of the wild type for both physiological substrates (i.e., K_m_G6P = 38.4 ± 4.1, K_m_NADP^+^ = 6.1 ± 1.2 μM, respectively), whereas the *k*_cat_ decreased near 50% for both mutants compared to the WT G6PD enzyme (Table 2).

The affinity for both physiological substrates in the double mutant G6PD A− was similar to that obtained for the G6PD Nefza variant, and was lower than the G6PD A+ variant. These results suggest that the single mutation of Leu→Pro 323 (G6PD Nefza) has a major contribution to the loss of affinity for both substrates in the double mutant G6PD A−.

Furthermore, the Class III mutants included in this study, G6PD A+ (114 ± 3.2 s^−1^), G6PD Nefza (126 ± 2.8 s^−1^), and one double mutant G6PD A− (35.8 ± 3 s^−1^) showed lower catalytic constant (*k*_cat_) values when compared with WT G6PD (i.e., 230 ± 7.6 s^−1^) (Table 2). The double mutant G6PD A− (Class III) showed a dramatic decrease in catalytic efficiency of 85%, while both Class III G6PD A+ and Nefza single mutants had a decrease of 50%, in relation to WT G6PD. The degrees of enzymatic dysfunction detected in the three clinical mutants were in accordance with the severity of the clinical manifestations, where the single natural Class III G6PD Nefza variant showed antecedents of hemolytic anemia due to bean consumption; while the in the double mutant G6PD A− classified as a Class III variant, the patients have showed episodes of hemolysis triggered by infections, drugs, or food was observed [12]. Although the double mutant G6PD A− has been classified as a Class III mutant according to hematological parameters of the patients, the recombinant G6PD variant showed a loss of catalysis similar with the previous values obtained for Class I mutants (Zacatecas, Durham, Nashville, Volendam and Andalus), which was near 75% with respect to WT [16,17,18]. However, these Class I mutants are related with severe clinical manifestations as chronic nonspherocytic hemolytic anemia (CNSHA) or with antecedents of neonatal jaundice and hemolytic crisis as is the case in the G6PD Zacatecas.

The alterations in specific activity obtained for the proteins and the low purification yield for the G6PD mutants were consistent with an important reduction in catalytic efficiency for the three clinical G6PD variants, which correlated with the reduced enzyme activity observed in the patients. These data indicated that catalytic efficiency was affected by the mutations and that the extent of influence both in the catalysis and the affinity for the two physiological substrates depended on the location of the mutation in the tertiary structure (Figure 1).

### 2.3. Evaluation of Protein Stability

#### 2.3.1. Thermostability Analysis

**Figure 5:**
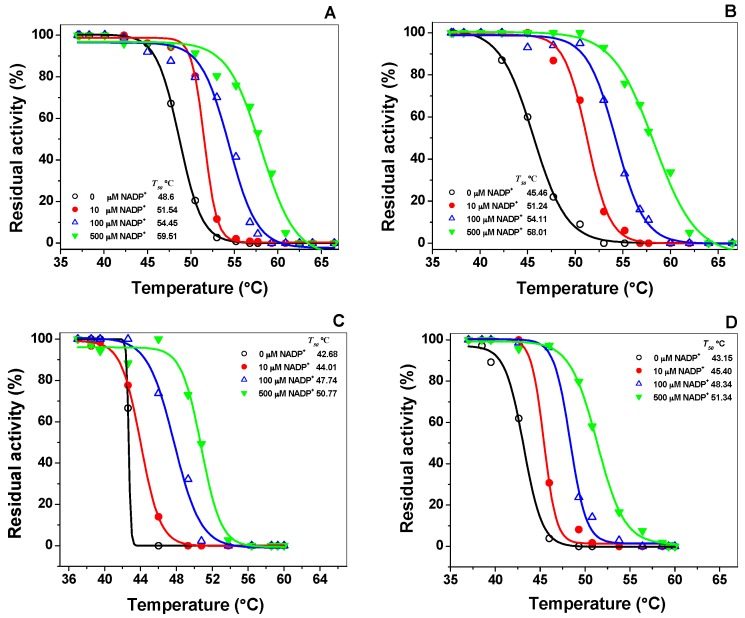
Thermal inactivation assays of recombinant G6PD variants in the presence and absence of different concentrations of NADP^+^. (**A**) WT G6PD; (**B**) G6PD A+; (**C**) G6PD Nefza; (**D**) G6PD A−. In all cases, 200 ng of total protein was used. Residual activity was expressed as a percentage of the activity for the same sample incubated at 37 °C. The assays were performed in triplicate; standard errors were lower than 5%.

Thermal inactivation assays have been widely used to evaluate the effect caused by the point mutation in the stability of the active site of the G6PD enzymes [19,20,21]. Stability of the active site of WT-G6PD and the effect of the three pathological mutants were analyzed with different concentrations of NADP^+^. Thermal inactivation assays for recombinant G6PD variants in the presence and absence of different concentrations of NADP^+^ and *T*_50_ (temperature at which the enzyme loses 50% of its original activity after incubation for 20 min) were performed. Figure 5 shows the *T*_50_ values for the WT G6PD enzymes and the three clinical mutants. In the absence of added NADP^+^, *T*_50_ values were 48.67 °C and 45.6 °C for the WT G6PD and G6PD A+ enzymes, respectively. As expected, G6PD Nefza and G6PD A− mutants were the most thermolabile proteins with *T*_50_ values of 42.6 °C and 43.1 °C, respectively; which were about 6 °C less with respect to those obtained for WT G6PD (Figure 5A–D). Furthermore, we also performed thermal inactivation assays in the presence of different concentrations of NADP^+^ (0–500 μM) and observed that with increasing concentrations of NADP^+^, the *T*_50_ values also increased for the three clinical mutants (Figure 5A–D).

Regardless of the location of these Class III mutations in the three-dimensional structure of the protein, we observed that the *T*_50_ values at 500 μM NADP^+^ for G6PD A+ variant was 12 °C higher when compared with the values obtained without NADP^+^. However, the G6PD Nefza and the double mutant G6PD A− were the least stable enzyme because it only increased by 8 °C when the NADP^+^ was added. It was interesting to note that the NADP^+^ dependent stabilization observed in the three clinical variants studied in this work was in concordance with the protective effect of NADP^+^ that has also been observed in the G6PD Yucatan [15], Mahidol [22], Andalus [16], Plymouth [22] and Viangchan [23,24] mutants, despite these mutations not being located near the active site or structural NADP^+^ binding in the native G6PD enzyme. The mutants analyzed in this study showed the protective effect of NADP^+^, which works as a stabilizer during thermal inactivation assays. The WT-G6PD was more stable than the variants involved in this study, indicating that the single G6PD A+, Nefza, and the double mutant G6PD A− variants were structurally unstable compared to WT-G6PD enzyme.

#### 2.3.2. Thermal Stability of Recombinant Human G6PD Enzymes

**Figure 6:**
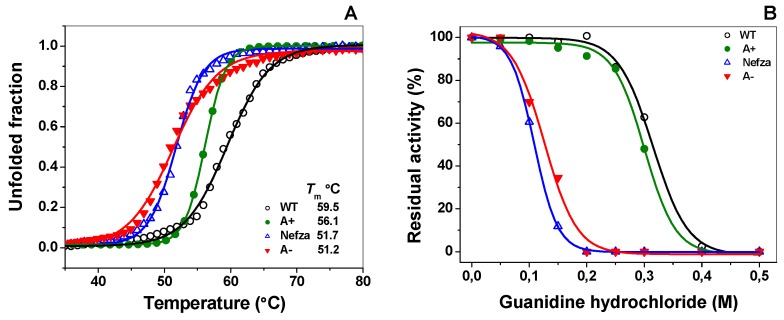
Stability analysis of recombinant human G6PD variants. (**A**) Thermal stability of G6PD variants. Changes in the CD signal at 222 nm were monitored as the temperature increased from 20 °C to 80 °C. The *T*_m_ (melting temperature midpoint of the transition values) was calculated for each variant. (**B**) Stability analysis of G6PD variants in the presence of guanidine hydrochloride (Gdn-HCl). All enzymes were incubated at 0.2 mg/mL in 50 mM phosphate buffer pH 7.35 in the presence of the indicated concentrations of Gdn-HCl for 2 h at 37 °C and subsequently the enzymatic activity was measured. Residual activity was expressed as a percentage of the activity for the same sample measured at 25 °C without Gdn-HCl and were diluted immediately before use. The experiments were performed in triplicate and the standard errors were less than 4%.

To gain insights on the effects of mutations on global structural stability, we determined the structural changes in G6PD variants when compared with the native enzyme using circular dichroism (CD) analysis at 222 nm when the temperature was changed. The temperature increases induced denaturation of all the proteins showing a two-state process and the temperature at which half of the secondary structure was unfolded was defined as *T*_m_. As shown in Figure 6A, the *T*_m_ for the WT G6PD enzyme (59.5 °C) was in concordance with those previously reported in References [15,18,23,24,25]. Thermal denaturation of the three clinical G6PD variants was different and dependent on the mutation site and in all cases exhibited a lower *T*_m_ when compared with the native enzyme. The change in the amino acid leucine 323 by proline that gives rise to G6PD Nefza exhibited a *T*_m_ value of around 51.7 °C, which represented 7.7 °C lower than that registered by the WT G6PD enzyme. The mutation in the amino acid asparagine 126 by aspartic acid (G6PD A+) showed a *T*_m_ value of 56.1 °C, which represented a more stable enzyme when compared with the G6PD Nefza. It should be noted that the double mutant G6PD A− was more thermally unstable than the two clinically single mutants (G6PD A+ and Nefza), with a *T*_m_ value of 51.2 °C. These results indicated that the alteration in the global stability of the G6PD A− protein was mainly due to the presence of the single mutation in the residue Leu323Pro (G6PD Nefza) and that the mutation in the amino acid Asn126Asp does contribute significantly to the alterations in the global stability of the double mutant G6PD A−. The changes observed in the structural stability of the single mutant G6PD Nefza and the double mutant G6PD A− might be related to the loss of catalytic efficiency of these enzymes (around 85% for Class III G6PD A− and 50% for the G6PD Nefza) and could have a relationship with the clinical manifestations in patients. Finally, it was striking to note that the *T*_m_ values obtained for the mutants Class III G6PD A− and G6PD Nefza were similar to the *T*_m_ values obtained for the Class I variants G6PD Wisconsin, Durham, and Nashville [16,24]. This indicated that this double G6PD A− mutant was as unstable as Class I G6PDs mutants, and that this double mutant must exhibit severe clinical manifestations, very similar to the Class I variants, despite being classified as a Class III variant.

#### 2.3.3. Stability of G6PD Variants in the Presence of Guanidine Hydrochloride (Gdn-HCl)

To evaluate the effect of the mutations in the structural stability of the recombinant human WT G6PD enzyme, we performed inactivation assays with or without the chemical denaturant guanidine hydrochloride (Gdn-HCl). This unfolding analysis has been used to determine conformational stability as the denaturing agent alters the tertiary structure of the protein where consequently catalytic activity is perturbed [18,23,26,27]. As shown in Figure 6B, the residual activity of WT G6PD and the three clinical mutants decreased when the concentration of Gdn-HCl was increased, and the *C*_1/2_ values (Gdn-HCl concentrations at which the enzymes lose 50% of original activity in 2 h at 37 °C) for each mutant was determined. The *C*_1/2_ values of Gdn-HCl observed for Class III G6PD A−, Nefza, and A+ mutants were 0.1, 0.12, and 0.2 M, respectively. As expected, the WT G6PD was the most stable protein in the presence of Gdn-HCl. Moreover, we observed that the single mutant Class III G6PD A+ was more resistant than the G6PD Nefza and G6PD A−, but was more susceptible than WT GPD; however, the single and double mutant Class III G6PD Nefza and A− were more susceptible to Gdn-HCl as they lost about 100% of their activity even after incubating with 0.2 M Gdn-HCl for 2 h. As for the thermal stability assays, we found that the *C*_1/2_ values obtained in these Class III G6PDs mutants were close to those previously reported in the Class I G6PD Zacatecas where a *C*_1/2_ value of 0.1 for Gdn-HCl was determined. WT G6PD lost 50% of its original activity in the presence of 0.31 M of Gdn-HCl and this was in agreement with the results from a previous report on WT G6PD [18,23]. These results and the other functional analyses described above, suggest that the single G6PD Nefza mutant was probably mainly responsible for the loss of catalytic activity and structural stability in the double mutant G6PD A− and that the G6PD Nefza and G6PD A− mutants were low stable enzymes with a decreased conformational stability when compared with the WT G6PD enzyme.

### 2.4. Spectroscopic Characterization

#### 2.4.1. Circular Dichroism (CD) Analysis

**Figure 7:**
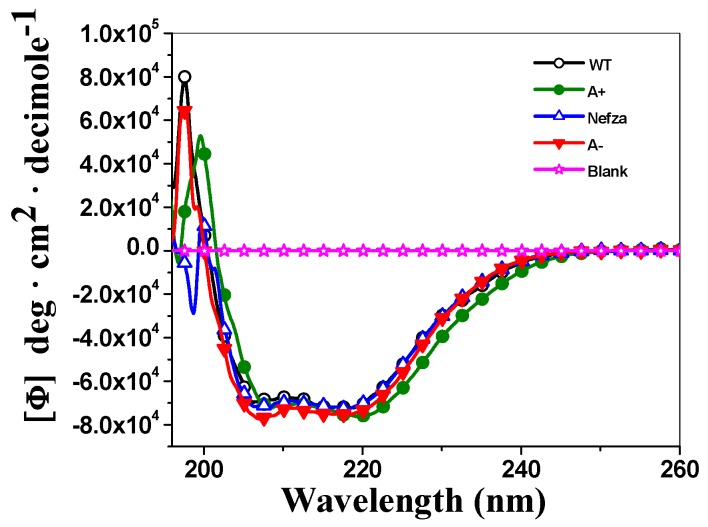
Structural characterization of recombinant human G6PDs enzymes. Circular Dichroism (CD) spectra in the far-UV region of recombinant human G6PD variants. The experiments were performed in triplicate; standard errors were less than 4%.

To determine if the diminished purification yield, the decrease in catalytic efficiency, and structural protein instability of all the variants enzymes were due to the disruption of the secondary structure of these enzymes, we evaluated the effect of these mutations by circular dichroism (CD) analysis in the far UV region (190–260 nm). As shown in Figure 7, the secondary structures of WT G6PD and the three mutants of the protein in the far-UV region showed minimum absorption peaks at 208 and 220 nm (Figure 7), which was consistent with the α-β structure of the protein [19]. As shown in Figure 7, both the two single mutants G6PD A+ and Nefza, and the double mutant G6PD A− showed the same pattern and intensity of CD spectra with respect to WT-G6PD, indicating that the mutations did not cause an alteration to the secondary structure of the enzyme. The alteration in the catalytic activity observed in the double mutant G6PD A− was not due to alterations on the secondary structure of the protein, but most likely due to conformational changes at the global level of the three-dimensional structure of the protein.

#### 2.4.2. Intrinsic Fluorescence and 8-Anilinonaphthalene-1-Sulfonate (ANS) Binding Analysis

**Figure 8:**
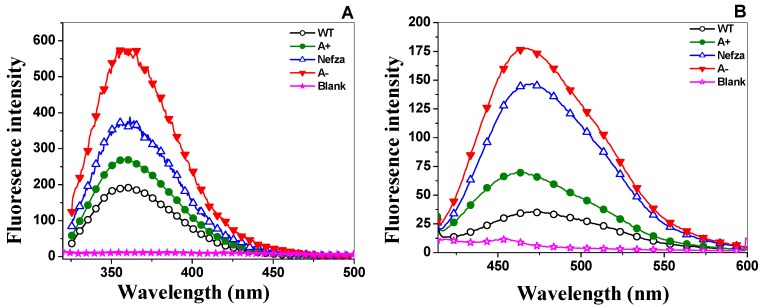
Fluorescence emission spectra of the G6PD variants. (**A**) Intrinsic fluorescence spectra of recombinant human WT G6PD and three clinical variants G6PD A+; Nefza and A− in the absence of NADP^+^; (**B**) 8-Anilinonaphthalene-1-sulphonate (ANS) accessibility assays of WT G6PD and the three clinical variants in the absence of NADP^+^. Values obtained from buffer containing ANS without protein (open stars) were subtracted from the recordings with protein. The experimental conditions for all the experiments are described in the Materials and Methods section.

To evaluate whether the activity loss had a correlation with the disruption of protein structural stability, we evaluated the intrinsic fluorescence and 8-anilinonaphthalene-1-sulfonate (ANS) analysis of the three clinical variants. As shown in Figure 8A, the fluorescence emission spectra for all variants showed an increased with respect to WT G6PD (Figure 8A). The fluorescence intensity for G6PD A+ was increased 1.6-fold compared to the WT G6PD enzyme, while the fluorescence intensity for Class III G6PD Nefza and for the double mutant G6PD A− was increased two-fold with respect to WT G6PD. However, it is very important to mention that the increase in intrinsic fluorescence intensity obtained for both for the single mutant G6PD Nefza and the double mutant G6PD A− mutant was also similar to the fluorescence obtained for both the Class I mutants G6PD Zacatecas and Durham, respectively, where the fluorescence intensity was increased two-fold [25]. This increase in intrinsic fluorescence intensity suggested modifications in the microenvironment of the tryptophan residues from a hydrophobic to a hydrophilic environment in the three-dimensional structure of this protein.

Finally, to corroborate if the changes observed in thermal denaturation, residual activity and intrinsic fluorescence of the single mutant G6PD Nefza and the double mutant G6PD A− were due to alterations in the global stability of the protein, we performed ANS assays that have been extensively used to monitor conformational changes in proteins [15,18,23,24,25]. The binding of ANS to the protein produces an increase in fluorescence intensity and is considered to be a suitable probe to monitor the changes in buried hydrophobic sites and electrostatic interactions of the protein residues. The results indicated that the fluorescence emission spectrum with ANS was 1.8-fold higher for the double mutant G6PD A− with respect to WT G6PD (Figure 8B), while the single mutant G6PD A+ and Nefza variants showed an increase of 1.2- and 1.4-fold in the fluorescence emission spectrum with ANS spectra with respect to WT-G6PD.

Alterations in the increase in fluorescence intensity and ANS fluorescence assays of the three clinical mutants of G6PD (A+, Nefza and A−) indicated conformational change in the three-dimensional structure and that ANS had access to buried hydrophobic pockets in the G6PD variant enzymes. Similar phenomena have also been observed in other G6PD variants such as G6PD Yucatan, Nashville, Durham, Zacatecas, Viangchan, Canton, and Mahidol [15,18,23,24,25]. It is striking to note that the single mutant G6PD Nefza provoked severe alterations in the three-dimensional structure with respect to WT G6PD, whereas the single mutant A+ showed only slight alterations with respect to WT G6PD. These results were in agreement with the data obtained from trials of expression, purification, steady-state kinetic parameters, thermal inactivation, circular dichroism, and thermal stability that the single mutant G6PD Nefza was responsible for the functional and structural alterations observed in the double mutant G6PD A−.

### 2.5. In Silico Mutagenesis and Computer Modeling

**Figure 9:**
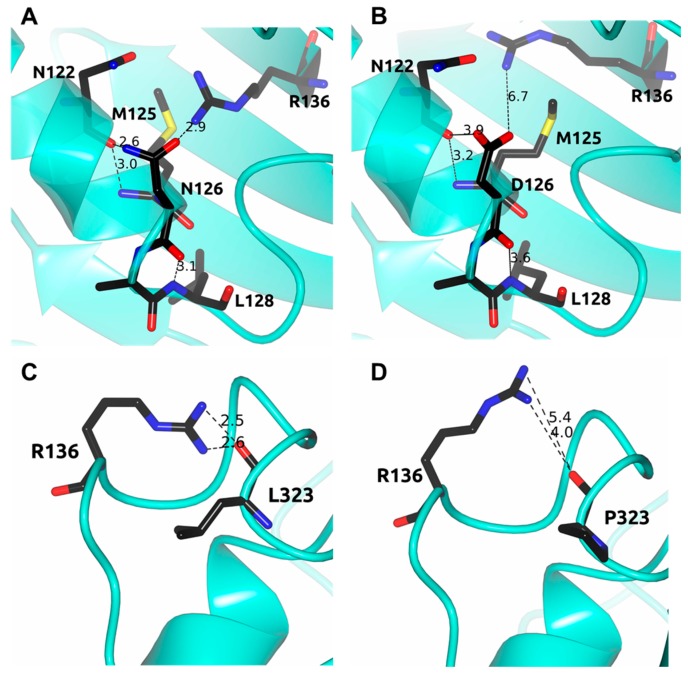
Structural comparison between the human G6PD enzyme (PDB entry 2BH9) and the minimized models of the Class III A+ and Nefza variants. (**A**) WT G6PD enzyme; (**B**) in silico N126D mutation; (**C**) WT G6PD enzyme; and (**D**) in silico L323P mutation. Residues are shown as black cylinders. Distances are in Å.

On the basis of the human G6PD three-dimensional structure, previous investigations have reported that amino acid mutations can explain altered biochemical function of G6PD. In this context, we performed in silico analysis for the three clinical mutants described in this work and in G6PD A+, when the amino acid asparagine was changed to aspartic acid at position 126, we observed that this mutation occurred in a α-helix exposed to a polar aqueous environment. The residual asparagine and aspartic acid showed a fairly similar low propensity to form α-helices. Furthermore, we observed that there was a strong interaction of 2.9 Å between the oxygen of the side chains of N126 with the side chain of R136. In addition, we observed an interaction between the nitrogen of the side chain of N126 and the oxygen of the carbonyl functional group of N122. Likewise, an interaction between the oxygen of the carbonyl group (peptide bond) of N126 with the nitrogen of the peptide bond of L128 and a fourth interaction between the carbonyl oxygen (peptide bond) of N122 and the nitrogen of the peptide bond of M125 were observed in the WT G6PD enzyme (Figure 9A). When N126 was replaced by D126, we observed that all these interactions weakened (increase the distance of the interaction) in mutant A+ (N126D) (Figure 9B), flexibilizing the zone, which could cause the enzyme to destabilize and lose 50% of catalytic activity even though this mutation is far from the active site or from the substrate- and structural NADP^+^ binding sites and are not part of the dimer/tetramer interface.

Regarding the G6PD Nefza variant, when we performed the in silico mutagenesis of leucine to proline in amino acid position 323, we observed that steric hindrance should be generated. Furthermore, we observed that an interaction between Leu323 and Arg330 was lost as the oxygen of Leu323 in the WT G6PD interacted with two nitrogen atoms of Arg330 (Figure 9B), and when the amino acid Leu323 was changed by Pro, the interaction was lost. This loss of interactions between the amino acids was probably the main cause of G6PD Nefza enzyme losing catalysis and structural stability affecting the conformation of the protein and causing the disease phenotype. We observed that in both single mutants (Class III G6PD A+ and G6PD Nefza) although the point mutations were distant from the active site or the dimer interface, these mutants showed a loss of catalysis around 50% with respect to WT G6PD. These data were in concordance with the previously reported studies that have also demonstrated differential impact of mutation sites on the activity of G6PD variants. For instance, a milder effect of mutation was observed in G6PD variants such as G6PD Canton, Viangchan, and Mahidol, where the mutations were not part of the substrate and structural NADP^+^ binding sites, or were not close to the dimer-tetramer interface [17,22,23,24].

Furthermore, this single mutant G6PD Nefza (L323P) showed lower expression, purification levels, had more thermolabile protein and in the three-dimensional structure was to some extent open, and presented exposed hydrophobic regions with respect to the WT G6PD enzyme. This phenomenon could increase instability, thus affecting catalysis and consequently the global stability of the protein. We believe that the biochemical changes observed in the single mutant G6PD Nefza were due to these alterations in the three-dimensional structure of the protein, since by itself this mutant presented the same parameters as the double mutant G6PD A−, which showed a reduction in thermostability in the absence or presence of NADP^+^, which could explain the reduced enzyme activity (from 10–20%) observed in the red blood cells of G6PD deficient individuals when compared with G6PD B^+^. Although the double G6PD mutations was not close to the structural NADP^+^ binding site or substrate binding site and were not part of the dimer-tetramer interface, they could also contribute to severe clinical phenotypes by different mechanisms and give rise to clinical manifestation as episodes of hemolysis triggered by infections, drugs, or food.

## 3. Materials and Methods

### 3.1. Construction of Recombinant G6PD by Site-Directed Mutagenesis

Three clinical variants, two single mutants G6PD A+, G6PD Nefza and the double mutant G6PD A− were generated by site-directed mutagenesis using the recombinant plasmid pET-HisTEVP-*g6pd* containing the full human *G6PD* gene (NM_001042351 access) as a template [28]. Specific mutagenic forward and reverse primers were designed to create the desired *G6PD* gene mutations (Table 3). Two flanking primers (Table 3) that contained *Nde*I and *Bpu11021* restriction sites (underlined) at the 5′ and 3′ ends, respectively, were used for polymerase chain reaction (PCR) amplification of the human *G6PD* gene. The PCR procedure was carried out according to Gómez-Manzo et al. [15,28].

PCR products were analyzed by 1% agarose gel electrophoresis and amplicons of the expected size (1545 bp) were purified and ligated into the pJET 1.2 vector (CloneJET PCR Cloning Kit; Thermo Scientific, Hudson, NH, USA). Each construction was amplified in competent *E. coli* BW25113 cells as previously reported in References [15,23,25]. Bidirectional DNA sequencing with verified internal forward and reverse sequencing primers were used to confirm that desired recombinant plasmids were obtained [15]. Verified sequences were digested with the restriction enzymes *Nde*I and *Bpu11021*, and sub-cloned into the pET-3a plasmid (Novagen, Madison, WI, USA) and used to transform competent *E. coli* BL21(DE3)Δ*zwf*::*kanr^r^* [15]. Oligonucleotide synthesis and DNA sequencing was provided by the Unidad de Síntesis y Secuenciación de ADN of Instituto de Biotecnología at the Universidad Nacional Autónoma de México.

### 3.2. Expression and Purification of Recombinant Human G6PD Enzymes

Expression of the G6PD variants was performed as previously described in References [15,23,25]. The optimal conditions for G6PD soluble protein expression of each mutant were obtained using 50 mL Luria Bertani (LB) culture medium with three expression temperatures and three isopropyl-β-d-thiogalactopyranoside (IPTG) concentrations, respectively. The samples were harvested at different times (2, 6, 12, and 18 h time courses), suspended in lysis buffer, and broken down by sonication [15,23,25]. The crude extract was clarified by centrifugation and aliquots from the supernatant were used to calculate specific G6PD activity.

The cells obtained under the best conditions for each G6PD mutant expression were used to inoculate 2 L of fresh LB medium 100 μg/mL of *Amp^R^* and *Kan^R^*, respectively, and grown at 37 °C with 200 rpm shaking. When the absorbance at 600 nm (OD_600_) reached 0.6–0.8, the cultures were induced with IPTG and grown up using the optimal expression conditions for each mutant. The cells were centrifuged, suspended in lysis buffer, and disrupted by sonication and the purification procedure for all the variants were performed as previously described in References [15,23,25] using 2′,5′-ADP Sepharose 4B affinity and anion exchange Q-Sepharose-4B columns (Sigma-Aldrich, St. Louis, MO, USA). The purity of the three recombinant enzymes was confirmed on 12% SDS-PAGE gels revealed with colloidal Coomassie Brilliant blue (R-250) (Sigma-Aldrich). The protein concentration was determined by the Lowry assay [30] using bovine serum albumin as the standard. The proteins were then preserved in ultra-pure glycerol (Sigma-Aldrich) at −70 °C.

### 3.3. Measurement of Steady-State Kinetic Parameters

The enzymatic activity of WT G6PD and the three clinical variants were determined spectrophotometrically by monitoring the reduction of NADP^+^ at 340 nm [15,18,23,24,25]. The standard activity assay was performed in a cuvette with a final volume of 1 mL and the reaction was initiated with the addition of 200 ng/mL of each variant enzyme. The standard reaction mixture contained 100 mM Tris-HCl buffer at pH 8.0, 3 mM MgCl_2_, 1 mM of glucose-6-phosphate, and 1 mM NADP^+^. Initial velocity data were obtained by varying one substrate (2.5 to 200 μM), while the second substrate was fixed at a saturating concentration. The initial velocity obtained for each concentration was used to calculate the rate of product formation of NADPH (μmol/min/mg) using the extinction coefficient of reduced nicotinamide adenine dinucleotide phosphate (NADPH) at 340 nm (6220 M^−1^·cm^−1^). The steady-state kinetic parameters, K_m_, *k*_cat_, and *V*_max_ were obtained by fitting the data to the Michaelis–Menten equation by non-linear regression calculations. One international unit (IU) of G6PD activity was the amount of enzyme required to produce 1 μmol of NADPH per minute under the assay conditions.

### 3.4. Evaluation of Protein Stability

#### 3.4.1. Thermal Inactivation Analysis

For thermal inactivation analysis, structural NADP^+^ was removed from the purified enzyme by buffer exchanged with 50 mM potassium phosphate buffer pH 7.35 containing 2 mM MgCl_2_ and enzyme concentration was subsequently adjusted to 0.2 mg/mL for each variant. The G6PD enzymes were incubated with varying concentrations of NADP^+^ (0, 10, 100, and 500 μM) for 20 min at different temperatures ranging from 37–60 °C as previously reported in References [15,18,23,24,25]. Samples were cooled down to 4 °C in a Thermocycler (MaxiGene Gradient, Axygen, Tewksbury, MA, USA) and the residual activity of the enzyme was determined and expressed as a percentage of the activity of the same enzyme incubated at 25 °C. All thermal inactivation tests were performed in triplicate.

#### 3.4.2. Thermal Stability of Recombinant Human G6PD Enzymes

Thermal stability and unfolding of WT G6PD and mutants were determined following changes by CD signal at 222 in temperature scans ranging from 20–90 °C at a rate of 1 °C/2.5 min increases. Structural NADP^+^ was removed from the purified enzymes as previously described and the concentration of G6PD was adjusted to 0.8 mg/mL [15]. The spectra of blanks were subtracted from those that contained the recombinant human WT G6PD enzyme and mutants, respectively. The fraction at which 50% of the protein was folded and unfolded was expressed as the melting temperature (*T*_m_) value and calculated for each G6PD variant.

#### 3.4.3. Stability of G6PD Variants in the Presence of Guanidine Hydrochloride (Gdn-HCl)

The stability of WT G6PD and mutant proteins in the presence or absence of Gdn-HCl was assessed as follows. Purified WT G6PD and mutant proteins free of bound NADP^+^ were adjusted to an enzyme concentration of 0.2 mg/mL. The samples were incubated at physiological temperature (37 °C) for 2 h in the presence of varying concentrations of Gdn-HCl (0.05, 0.10, 0.15, 0.20, 0.25, 0.30, 0.40, and 0.5 M). The residual activity of the enzyme was measured and expressed as a percentage of the activity of the same enzyme incubated at 37 °C in the absence of Gdn-HCl. The experiment was performed in triplicate.

### 3.5. Spectroscopic Characterization

#### 3.5.1. Circular Dichroism (CD) Analysis

Secondary structure of the G6PD variants was evaluated spectroscopically by circular dichroism (CD) in a spectropolarimeter (Jasco J-810^®^, Easton, MD, USA) equipped with a Peltier thermostated cell holder in a 1 mm path-length quartz cuvette as previously described in References [15,18,23,24,25]. Far UV-CD spectra of the G6PD variants were detected from 200–260 nm at 1 nm intervals using a protein concentration of 0.8 mg/mL (in the absence of NADP^+^) in 50 mM phosphate buffer at pH 7.35. The experiments were performed in triplicate at 25 °C.

#### 3.5.2. Intrinsic Fluorescence and 8-Anilinonaphthalene-1-Sulfonate (ANS) Binding Analysis

Conformational tertiary structure changes in the G6PD enzymes were evaluated by intrinsic fluorescence and their capacity to bind 8-anilinonaphthalene-1-sulfonate (ANS) assays. Protein fluorescence spectra from 310–500 nm were obtained at 25 °C in a Perkin-Elmer LS-55 fluorescence spectrometer (Perkin Elmer, Wellesley, MA, USA) using excitation at 295 nm, with excitation and emission slits of 4.5 and 3.7 nm, respectively. Assays were conducted in a quartz cell with a path length of 1 cm and a protein concentration of 0.1 mg/mL in 50 mM phosphate buffer pH 7.4.

ANS assays were performed in 25 mM phosphate buffer, pH 7.4 at 25 °C in a Perkin-Elmer LS 55 fluorescence spectrometer, using an excitation wavelength of 395 nm and recording emission spectra from 400–600 nm with excitation and emission slits of 10 nm, respectively. The final concentrations of ANS and the G6PD were 165 μM and 400 μg/mL, respectively. Background fluorescence from the buffer (blank), and buffer plus ANS was subtracted from those that contained the respective protein [15,23,25]. Both assays were performed in triplicate.

### 3.6. In Silico Mutagenesis and Modeling

Mutations in the crystal structure of human G6PD (PDB entry 2BH9) at positions 126 and 323 were generated in silico using the standard rotamer library of Coot [31]. The mutant models were subjected to energy minimization using freely available YASARA software (Vienna, Austria) [32]. The graphical representations were prepared with CCP4 mg version 2.10.4 [33].

## 4. Conclusions

In conclusion, we characterized the effects of two single G6PD mutations, G6PD Nefza and G6PD A+, clinical mutations, and the double mutant generated by the combination of both (G6PD A−), all classified as Class III variants. Analysis of the mutation effects and relation to disease severity was performed using different structure and protein dynamic approaches. We found that mutations caused perturbation in the activity and structural stability of the whole enzyme instead of limiting the effects to the local active site region. We also demonstrated that the main alterations in catalytic activity and structural modifications in the double G6PD A− mutant were due to the presence of the single G6PD Nefza mutant. More importantly, our study showed that biochemical and structural changes found in G6PD Nefza and A− variants matched those reported for Class I G6PD variants, suggesting the need to re-classify these mutants which should include clinical and biochemical characteristics of these G6PD variants. Finally, from the solved three-dimensional structure of the human G6PD protein, we defined changes in the interactions of the amino acid that offer a molecular explanation for the effects of these mutations, and provide a molecular explanation for clinical manifestations observed in individuals with G6PD mutations.

## Figures and Tables

**Table 1:** Purification summary of three recombinant human G6PD enzymes.

G6PD	Total Protein (mg)	Specific Activity (IU·mg^−1^)	Total Activity (IU)	Yield (%)
**WT**	4.8	224	1075	61
**A+**	3.6	114	410	43
**Nefza**	3.52	62	218	28
**A−**	3.53	22	77	24

Values are for a typical expression purification experiment; the result varied <10% from batch to batch. The G6PD activity was measured under the standard conditions described in the Materials and Methods.

**Table 2:** Steady-state kinetic parameters of human recombinant G6PDs proteins.

Kinetic Constants	WT-G6PD	Mutants		
		A+	Nefza	A−
K_m_·G6P (μM)	38.4 ± 4.1	56.4 ± 5.5	50.4 ± 6.2	33.8 ± 3.5
K_m_·NADP^+^ (μM)	6.1 ± 1.2	12.9 ± 1.4	16.4 ± 1.6	14.3 ± 2.1
*k*_cat_ (s^−1^)	230 ± 7.6	114 ± 3.2	126 ± 2.8	35.8 ± 3
*k*_cat_/K_m_·G6P (μM^−1^·s^−1^)	5.9 ± 0.6	2.0 ± 0.1	2.4 ± 0.2	1.1 ± 0.1
*k*_cat_/K_m_·NADP^+^ (μM^−1^·s^−1^)	37.3 ± 3.1	8.7 ± 0.7	7.5 ± 0.6	2.5 ± 0.2

The parameters in each case were obtained from three independent experiments and from different enzyme preparations.

**Table 3:** List of primers used in this study.

**Strain *E. coli***	**Relevant Characteristic(s) or Sequence**	**Source and/or Reference**
BW25113	F^−^, DE(araD-araB)567, lacZ4787(del)::rrnB-3, LAM^−^, rph-1, DE(rhaD-rhaB)568, hsdR514	[29]
BL21(DE3)Δ*zwf*::*kan^r^*	F^−^ ompT gal dcm lon hsdS_B_(r_B_^−^ m_B_^−^) λ(DE3 [lacI lacUV5-T7 gene 1 ind1 sam7 nin5]) Δ*zwf-777*::*kan*.	[15]
**Plasmids**		
pET-HisTEVP-*G6PD*	pETg6pd carrying the human *G6PD* gene, *Amp^R^*	[28]
Pjet*G6PD* A+	pJET 1.2 plasmid carrying the human *G6PD* gene with a Asn126Asp mutation in the G6PD protein, *Amp^R^*	This study
Pjet*G6PD* Nefza	pJET 1.2 plasmid carrying the human *G6PD* gene with a L323P mutation in the G6PD protein, *Amp^R^*	This study
pJET*G6PD* A−	pJET 1.2 plasmid carrying the human *G6PD* gene with a double mutation of Asn126Asp + Leu323Pro in the G6PD protein, *Amp^R^*	This study
pET*G6PD* A+	pET-3a carrying the human *G6PD* gene with a Asn126Asp mutation in the G6PD protein, *Amp^R^*	This study
pET*G6PD* Nefza	pET-3a carrying the human *G6PD* gene with an Leu323Pro mutation in the G6PD protein, *Amp^R^*	This study
pET*G6PD A−*	pET-3a carrying the human *G6PD* gene with a double mutation of Asn126Asp + Leu323Pro in the G6PD protein, *Amp^R^*	This study
**Mutagenesis**	**Primer Sequence**	
A+ forward	5′-AGCCACATGGATGCCCTCCAC-3′	This study
A+ reverse	5′-GTGGAGGGCATCCATGTGGCT-3′	This study
Nefza forward	5′-TCCACCAACCCAGATGACGT-3′	This study
Nefza reverse	5′-ACGTCATCTGGGTTGGTGGA-3′	This study
**Oligonucleotides for sequencing**	**Primer Sequence**	
Flanking *NdeI* forward	5′-CGACAGC**CATATG**GCAGAG-3′	This study
Flanking *Bpu* reverse	5′-TGC**GCTGAG**CTCAGAGCTT-3′	This study
Internal G6PD forward	5′-GGCCAACTGCCTCTTCTAC-3′	This study
Internal G6PD reverse	5′-GAGAAGGTCAAGATGTTGAAATG-3′	This study

The locations of the mutagenic oligonucleotides are in bold.
